# Macrophage activation syndrome and COVID-19

**DOI:** 10.1186/s41232-020-00131-w

**Published:** 2020-08-06

**Authors:** Ryo Otsuka, Ken-ichiro Seino

**Affiliations:** Institute for Genetic Medicine, Hokkaido University, Kita-15, Nishi-7, Sapporo, Hokkaido 060-0815 Japan

**Keywords:** COVID-19, SARS-CoV-2, Macrophage activation syndrome, Cytokine storm, Anti-cytokine therapy

## Abstract

An emerging, rapidly spreading coronavirus SARS-CoV-2 is causing a devastating pandemic. As we have not developed curative medicine and effective vaccine, the end of this life-threatening infectious disease is still unclear. Severe COVID-19 is often associated with hypercytokinemia, which is typically found in macrophage activation syndrome. SARS-CoV-2 infection causes this strong inflammation within the lung and propagates to respiratory and, ultimately, systemic organ malfunction. Although we have not fully understood the physiological and pathological aspects of COVID-19, current research progress indicates the effectiveness of anti-cytokine therapy. Here, we summarize macrophage activation syndrome and its possible contribution to COVID-19, and cytokine targeted attempts in severe COVID-19 cases.

## Introduction

Cytokine storm is a status of the immune system in which various immune cells are extremely activated and produce large amounts of cytokines, then, in turn, exhibit systemic hyperinflammatio n[1]. It often confers multiple organ failure and a high mortality rate. Various inflammatory cytokines or chemokines such as tumor necrosis factor (TNF)-α, type I and II interferons (IFNs), interleukin (IL)-1, IL-6, CCL2, or monocyte chemotactic protein-1 (MCP-1), as well as immunosuppressive cytokines such as IL-10 or transforming growth factor-β, have been implicated. Similarly, various immune cells such as T cells, B cells, dendritic cells (DCs), or macrophages are important to understand the pathophysiology of cytokine storm. Among those, activation of macrophages has been particularly paid attention, as it is especially called macrophage activation syndrome (MAS) [2]. MAS has been suggested to be also involved in the etiology of hyperinflammatory responses in the course of treatment with chimeric antigen receptor T cell for leukemic patients.

Cytokine storm has been observed and discussed in various clinical conditions such as rheumatological or hematological disorders [2]. Furthermore, it sometimes occurs in infectious diseases and elicits a refractory condition against intensive therapies. It is related with the induction of acute respiratory distress syndrome (ARDS), which is one of the severest pathological status of respiratory systems, causing pulmonary edema, decreased gas exchange, and fatal hypoxia [3].

Recently, it has been suggested that cytokine storm, particularly MAS, is involved in coronavirus disease 2019 (COVID-19)-associated pneumonia and its exacerbation [4]. Although the major body of COVID-19 patients shows none to mild pulmonary symptoms, approximately 20% of patients show severe pulmonary dysfunction. Among those, a certain percentage of patients undergo life-threatening, critical pneumonia, the treatment for which extracorporeal membrane oxygenation is required. The reason why only a part of severe acute respiratory syndrome coronavirus 2 (SARS-CoV-2)-infected patients show such severe inflammatory condition has not been clarified. Still, it is possible that the causative virus for COVID-19, SARS-CoV-2, infect with particular types of cells such as endothelial vessels in the lung, or alveolar wall or macrophages. The infection to the cell types may induce immune responses leading to the cytokine storm, including MAS.

In this brief review, we discussed a possible involvement of MAS in the pathophysiology of COVID-19, especially in cases with severe inflammatory pneumonia.

## An overview of MAS and possible therapies

MAS is a state of systemic hyperinflammation and often be observed in patients with infections, malignancy, or pediatric rheumatological diseases, such as systemic juvenile idiopathic arthritis (SJIA) [2]. MAS is typified by markedly upregulated expression of pro-inflammatory cytokines, which is called “cytokine storm.” Without any therapeutic intervention, this strong inflammation results in severe tissue injury and, ultimately, patient death. Several research pieces have revealed the involvement of particular cytokines in this phenomenon, especially TNF-α, IL-6, and IL-1β [5, 6]. Macrophages in MAS state produce a high amount of these pro-inflammatory cytokines upon stimulation. Billiau et al. reported the histopathological evidence that macrophages in the liver of patients suffering from MAS were expressing TNF-α and IL-6 [7]. Together with IL-1, TNF-α and IL-6 trigger a cascade of inflammatory pathways that synergistically activate and augment inflammation [8]. Thus, serum levels of these cytokines are often at a high level in MAS patients [5]. Inflammation is known to destruct the precise balance between coagulation and fibrinolysis. Certain inflammatory cytokines such as TNFα and IL-1 initiate tissue factor production from monocytes and macrophages [9], leading to the activation of coagulation, while IL-1 and IL-6 increase the production of plasminogen activator inhibitor [10]. Thus, the overproduction of inflammatory cytokines along with MAS also promotes intravascular coagulation. Standard treatment for MAS includes several immunosuppressive drugs, such as steroids, calcineurin inhibitors, or anti-thymocyte globulin [5]. In spite of such broad immunosuppression, it is difficult to mitigate severe MAS symptoms. Therefore, previous researches have spent their efforts on the pursuit of finding a new therapeutic target. In this context, cytokines highly produced in MAS patients are potential candidates, and some clinical reports provided promising results by cytokine-targeting therapy.

MAS occurs around 10% of SJIA, a systemic inflammatory disorder of non-particular etiology characterized by arthritis and systemic features [2]. A case report on a 27-year-old female SJIA patient was clinically diagnosed as MAS and presented an extremely high level of TNF-α in the serum [11]. In contrast, a remarkably low level of soluble TNF receptor (TNFR) was detected. Because soluble TNFR acts as an antagonist of TNF, these clinical parameters suggested overactivated TNF signaling as a cause of the hyperinflammation. Although the patient was utterly unresponsive to the series of treatment including steroid pulse and cyclosporine treatment, administration of the soluble TNFR etanercept dramatically improved the symptoms. Etanercept is a fusion protein of TNFR and immunoglobulin domain, which replaces the function of endogenous soluble TNFR. The case report showed complete remission of the patient and suggested a therapeutic potential of anti-TNF-α reagent. On the other hand, some researchers indicate totally opposite clinical observations that anti-TNF therapy with etanercept, in turn, triggers cytokine storm in a patient with systemic sclerosis and necrotizing fasciitis [12]. Together, we need more experimental knowledge to solidify the potential of TNF-α to be a therapeutic target in MAS treatment.

Anti-IL-6 treatment with specific blocker tocilizumab has also been suggested effective in attenuating clinical symptoms of MAS. Shimizu et al. defined that patients with MAS showed significantly lower ferritin, CRP, triglycerol, fibrinogen, and aspartic aminotransferase serum levels when receiving tocilizumab treatment, indicating an alleviation of systemic inflammation [13]. However, the improvement in clinical and laboratory features of MAS may compromise MAS diagnosis. Their analysis also showed that some cases representing laboratory MAS features failed to fulfill clinical MAS criteria, resulting in only 20% of possible MAS patients meet the 2016 MAS classification. These backgrounds underlying anti-IL-6 therapy on MAS may preclude the precise estimation of the treatment outcome, and the specific effect of tocilizumab on MAS remains to be investigated.

A case report by a research group from the University of Miami Miller School of Medicine presented that IL-1 receptor antagonist anakinra showed promising results in MAS treatment [14]. IL-1 is a potent stimulator of IL-6 production from macrophages, and serum IL-1β is often at a high level among SJIA patients. The activation of the IL-1 receptor signaling pathway is also suggested by gene expression profiles of peripheral blood from SJIA patients with severe inflammation. Other studies also indicated promising results of anakinra treatment in MAS patients [15]. Although its direct contribution to the onset of MAS remains unclear, these observations predispose us to expect the therapeutic potential of IL-1 blockade.

Collectively, cytokine-targeted MAS therapy has been reported by various research groups. In addition to the studies mentioned above, CD28, JAK1/2, and IFN-γ were also implicated as potential therapeutic targets [16–18]. Particularly, when considering the cases of infection-induced MAS, these specific approaches may reduce major concerns accompanied by traditional therapy with immunosuppressants, which broadly suppress immune activation.

## MAS and ARDS in COVID-19

COVID-19-related hyperinflammation shares its clinical features with previously reported MAS symptoms. IL-1β, IL-2, IL-6, IL-7, IL-17, and TNF-α were reported to be highly upregulated in patients with severe COVID-19 pneumonia patients [8, 19]. Particularly, plasma levels of IL-2, IL-7, TNF-α, G-CSF, CXCL10, CCL3, and MCP1 were much upregulated in the patients treated in ICU compared to those in non-ICU [19]. Not only the hypercytokinemia but also the increased serum levels of ferritin, CRP, and D-dimer indicate the development of MAS-like severe inflammation and fibrinolysis in COVID-19 patients [20, 21]. Despite the above clinical features shared with classical MAS, some are not compatible with known MAS status. Hyperferritinemia is indeed a hallmark of COVID-19 pneumonia. One reported data showed that the median of serum ferritin concentration was about 800 ng/ml in the Wuhan patient cohort [22]; however, it is still lower than that of MAS, which often exceeds 10,000 ng/ml [23]. Moreover, MAS is accompanied by reduced fibrinogen and platelet count and increased D-dimer [5], which indicates systemic disseminated intravascular coagulation. Conversely, only a high D-dimer was typically found in COVID-19 patients [24]. Another regular feature of MAS is hepatosplenomegaly which was absent in reported COVID-19 patients [4, 24]. In sum, at least accumulating clinical evidence implies the coincidence of MAS-like hyperinflammation with COVID-19 pneumonia; however, its immunological and pathological manifestations were observed mainly in the lung, which is associated with ARDS.

There are various etiologies under the onset of ARDS. Clinically, ARDS is featured by hypoxemia, diffuse pulmonary infiltrates, pulmonary edema, and reduced lung compliance, collectively result in rapid respiratory failure [25]. In terms of plasma levels, TNF-α, IL-1β, IL-6, and IL-8 are elevated and show a higher concentration in non-survivors than survivors of ARDS patients [26]. It has been reported that approximately 20% of patients developed ARDS during the course of COVID-19 [27], with some cases rapidly worsened and died.

Clinical characteristics studied in a cohort of Jinyintan Hospital in Wuhan identified that 41.8% of COVID-19 patients developed ARDS [20]. The patients were diagnosed as ARDS immediately after their admission to the hospital. Of note, the study reported all of the patients’ death followed ARDS and mechanical ventilation. One of the cell types considered to play an essential role in ARDS onset is macrophage residents in alveolar [28]. Those alveolar macrophages would be activated upon lung infection and release pro-inflammatory cytokines such as TNF-α, IL-1β, and IL-6. Recent genomic studies revealed that angiotensin-converting enzyme 2 (ACE2) and transmembrane protease, serine 2 (TMPRSS2), expressed on the surface of type II pneumocytes, would mediate SARS-CoV-2 entry into target cells [29, 30]. Viral RNA invasion into the target cells elicits the production of pro-inflammatory cytokines via the activation of NF-κB pathway [31]. Besides, SARS virus infection has been reported to induce pyroptosis, a state of cell death mediated by activation cascade of NLRP3 inflammasome [32, 33]. One downstream indicator of pyroptosis is IL-1β; the evidence of high serum IL-1β may be one indicative of pyroptosis in COVID-19-related lung inflammation. Pyroptosis results in releasing damage-associated molecular patterns, which stimulate neighboring macrophages to produce pro-inflammatory cytokines and chemokines. The macrophages receiving inflammation signals, in turn, recruit immune cells such as T cells into the site of inflammation. Additionally, viral RNA entry into macrophages also provokes macrophage activation [34]. Although macrophages do not express ACE2 and TMPRSS2, alveolar macrophages may uptake viral RNA through phagocytosis and the degradation of virus-infected cells. Otherwise, antibodies to SARS-CoV-2 virus opsonize virus particles and allow macrophages to engulf viruses via Fc-receptor-mediated endocytosis. Indeed, previously analyzed SARS virus was detected in the cytoplasm of alveolar macrophages [35].

**Fig. 1:**
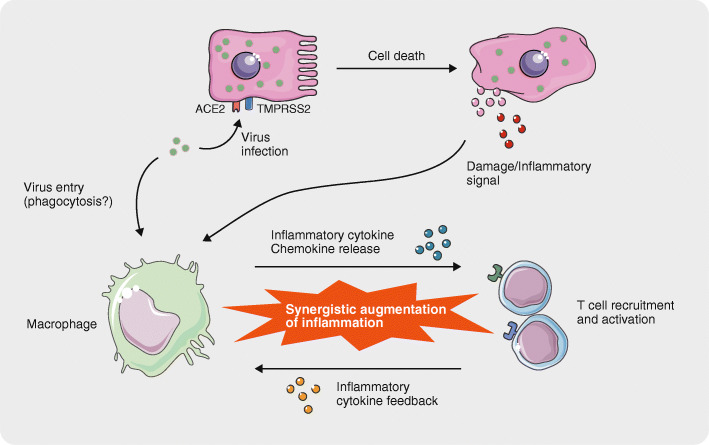
Macrophage activation and its synergistic augmentation of inflammation in COVID-19. SARS-CoV-2 infects pneumocytes or enters macrophage. Infection-induced cell death promotes the release of damage signal molecules and inflammatory cytokines. Macrophages are activated upon inflammatory signal or viral genome entry then produce inflammatory cytokines and chemokines which recruit and activate T cells. Feedback inflammatory signals from T cells further activate macrophages. Synergistic augmentation of inflammation spread the entire lung and consequently systemic organ

Thus, the commencement of local inflammation induced by SARS-CoV-2 infection activates macrophages at that site, spreading rapidly to the entire lung, possibly due to the abundant expression of virus entry receptors, ACE2 and TMPRSS2 [36]. Accumulation of immune cells accelerates the progression of lung inflammation into ARDS. In severe cases, local inflammation cannot be sedated within the lung and, consequently, propagates to multiple organ failure and death (Fig. 1).

## Therapeutic potential of conventional MAS therapy for COVID-19

As COVID-19 patients represent symptoms resembling MAS, it is of our great interest to know whether anti-cytokine reagents used in MAS are also effective in COVID-19 treatment. A report of the preliminary trial by the University of Science and Technology of China suggests the effectiveness of tocilizumab in severe COVID-19 cases [37]. They report an immediate decrease in CRP and body temperature and improvement of peripheral oxygen saturation. It is noteworthy that no serious adverse effects were observed in all patients, and after tocilizumab treatment, 90% of the patients were discharged from the hospital within 3 weeks. A more recent study of 100 COVID-19 patients from Italy also demonstrated the rapid improvement from severe ARDS condition by tocilizumab treatment [38]. The therapeutic potential of blockade of IL-6 signaling for COVID-19 is reviewed in more detail in other review articles in this series.

Cavalli et al. from Vita-Salute San Raffaele University reported the cases of COVID-19-associated ARDS patients subjected to anakinra treatment [39]. They showed significant improvement in patients’ outcomes, which was supported by 90% survival in high-dose anakinra-treated patients while 56% survival in the standard treatment group at the study endpoint. The rate of ventilation-free survival was also higher, albeit not statistically significant, in anakinra-treated patients. As there are yet a small number of cases reporting the efficacy of anakinra in severe COVID-19 patients, it is expected to compile additional clinical evidence. An up-to-date study indicates that the inhibition of Bruton tyrosine kinase (BTK) by acalabrutinib effectively improved COVID-19 patient outcomes [40]. BTK is involved in NLRP3 inflammasome activation cascade, which subsequently triggers IL-1β production [41]. As IL-1β signal inhibition by anakinra treatment showed a favorable outcome, BTK targeted therapy may also be a prospective therapeutic option for COVID-19.

Although the abovementioned clinical trials were uncontrolled ones, the effects of tocilizumab and anakinra seem promising. Currently, we can find more than 40 clinical trials for surveying an efficacy of anti-IL-6 receptor antibody tocilizumab and 15 trials on anakinra, either alone or combined with other drugs (https://clinicaltrials.gov). Additionally, TNF, IFN-γ, and GM-CSF are also targeted in other clinical trials. These trials are at least in part related with MAS, because the targeted cytokines have been highly implicated with MAS and macrophage functions. COVID-19-related hyperinflammation is only found in part of entire patients; however, no specific treatment for this population has been reported. Thus, further studies to discover an optimal treatment are urgently needed. Extensive examination of MAS status in COVID-19 may help discover new targets.

## Conclusion

SARS-CoV-2 is threatening millions of lives worldwide. Due to the recent rapid progress of understanding the nature of the novel spreading virus, we are getting be able to prevent its massive spread in particular areas. However, we have not yet reached the curative medicine or effective vaccine. Severe cases of COVID-19 are often observed with ARDS, representing the MAS-like clinical and laboratory features. Previous studies have revealed that MAS symptoms can be ameliorated by anti-cytokine therapy. To date, anti-IL-6, IL-1, and TNF have shown promising outcomes in MAS treatment. Based on these findings, anti-IL-6 or IL-1 treatment was carried out in COVID-19 and showed significant improvement in the patients’ symptoms.

The world is confronting a difficult situation. Not only clinicians and researchers but also people all over the world expect the development of the best therapeutic agent to COVID-19. More than 1900 clinical studies are in progress across the globe, and they may offer clues on novel treatment.

## Data Availability

Not applicable

## Abbreviations

ACE2: Angiotensin-converting enzyme 2
ARDS: Acute respiratory distress syndrome
COVID-19: Coronavirus disease 2019
GM-CSF: Granulocyte macrophage-colony stimulating factor
IFN: Interferon
IL: Interleukin
MAS: Macrophage activation syndrome
MCP1: Monocyte chemotactic protein 1
SARS-CoV: Severe acute respiratory syndrome coronavirus
SJIA: Systemic juvenile idiopathic arthritis
TMPRSS2: Transmembrane protease, serine 2
TNF: Tumor necrosis factor
TNFR: Tumor necrosis factor receptor
